# HER2在NSCLC中的作用

**DOI:** 10.3779/j.issn.1009-3419.2015.10.08

**Published:** 2015-10-20

**Affiliations:** 100071 北京，中国人民解放军军事医学科学院附属医院肺部肿瘤科 Department of Lung Oncology, Affiliated Hospital of The PLA Military Academy of Medical Sciences, Beijing 100071, China

**Keywords:** 肺肿瘤, 人表皮生长因子受体2, 治疗, Lung neoplasms, HER2, Therapy

## Abstract

过去几年中, 随着分子靶向药物的引入, 非小细胞肺癌(non-small cell lung cancer, NSCLC)的药物治疗策略发生了巨大变化, 向基于组织学和分子水平的治疗手段转变。表皮生长因子受体(epidermal growth factor receptor, *EGFR*)突变、Kirsten鼠肉瘤(Kirsten rat sarcoma, *KRAS*)癌基因突变、间变淋巴瘤激酶(anaplastic lymphoma kinase, *ALK*)重排等的发现, 影响着NSCLC治疗的发展。最近, 对人表皮生长因子受体2(human epidermal growth factor receptor 2, HER2)研究重燃兴趣, 这一基因改变与NSCLC对不同酪氨酸激酶抑制剂(tyrosine kinase inhibitors, TKIs)的敏感性相关, 其具有可能的预测作用, *HER2*扩增可能是*EGFR*突变肿瘤对EGFR-TKIs获得性耐药的机制之一。其次, *HER2*突变可能阐明一条新的靶向治疗NSCLC的策略。本文将对NSCLC中HER2异常调节发挥的作用做一简要介绍。

## Abstract (en)

The therapeutic strategy of non-small cell lung cancer (NSCLC) is dramatically changed with the introduction of molecular targeted drugs in the last years, resulting in a series of results in histologic and molecular level.The discovery of epidermal growth factor receptor (*EGFR*) mutation, Kirsten rat sarcoma (*KRAS*) viral oncogene mutation and anaplastic lymphoma kinase (*ALK*) rearrangement, has profoundly influenced the development of treatment of NSCLC.Recently, there is a renewed interest in the human epidermal growth receptor 2 (HER2), where genetic alteration in NSCLC is associated with the different sensetivity of EGFR tyrosine kinase inhibitors (TKIs), to have a prognostic effect.*HER2* amplification in *EGFR* mutation tumors may become a mechanism of acquired resistance to the TKIs.Besides, *HER2* mutation may become a novel therapeutic strategy of NSCLC.

肺癌是目前为止死亡率最高的肿瘤^[1]^, 非小细胞肺癌(non-small cell lung cancer, NSCLC)占到了肺癌的80%^[2]^。过去几年中, 随着分子靶向药物的引入, NSCLC的治疗策略发生了巨大改变。BR.21试验与对照组相比, 表现出总体生存优势^[3]^。在存在表皮生长因子受体(epidermal growth factor receptor, EGFR)酪氨酸激酶结构域活性突变的患者中, EGFR酪氨酸激酶抑制剂(EGFR tyrosine kinase inhibitors, EGFR-TKIs)的使用已成为最优治疗方案^[4, 5]^。2004年在一小部分NSCLC患者中发现了*EGFR*突变, 随后发现了高反应性的EGFR-TKIs吉非替尼^[6, 7]^, 加速了NSCLC中其他激酶突变的研究。间变淋巴瘤激酶(anaplastic lymphoma kinase, ALK)重排的发现, 4年后美国食品药品管理局对克唑替尼快速审批^[8]^, 这些加速了一系列分子特异性抑制剂的研究。肺癌突变联盟的临床实验(NCT01014286)结果表明, 肺腺癌中Kirsten鼠肉瘤(Kirsten rat sarcoma, *KRAS*)突变占25%, *EGFR*突变占21%, *ALK*重排占8%, 这些患者似乎应该进行基因型定向治疗^[9]^。

自从并无对照的临床病例报道了人表皮生长因子受体2(human epidermal growth factor receptor 2, *HER2*)突变NSCLC患者服用抗HER-2药物的效果^[10]^, 一些靶向药物在*HER-2*突变患者身上进行评估, 对这个致癌因子的兴趣不断增加。本文将对NSCLC中HER2异常调节发挥的作用做一简要介绍。

## 1 HER2信号通路

HER家族是一个典型酪氨酸激酶受体家族, 由HER1(EGFR/ERBB1)、HER2(ERBB2)、HER3(ERBB3)、HER4(ERBB4)4个成员组成。每一受体都具有细胞外配体结合结构域、一个跨膜α螺旋以及细胞内酪氨酸激酶结构域。配体结合至HER细胞外结构域时, 将引起构象的改变从而产生二聚化。受体二聚化是HER发挥功能所必需的, 可以发生在两个相同或者不同的HER受体。配体的结合促进了受体的二聚化和胞浆尾部酪氨酸残基的自身磷酸化, 这将作为一系列具有SH2和PTB结构域的接头蛋白的停泊位点, 激活细胞内的信号级联反应, 包括MAPK和PI3K/AKt通路^[11-16]^。

虽然所有四种HER都具有同样的必需的结构域, 但每个结构域的功能活性并不相同。实际上, HER3能够结合若干配体却缺乏内在的酪氨酸激酶活性, 并且只能和其他HER发生异源二聚化。HER2具有酪氨酸激酶活性结构域却缺乏特异配基, 可以和其他HER很好地形成异源二聚体。他以广泛的“活性”构象状态存在, 使他能随时与已结合配体的家族受体相互作用。由于HER2的独特性质, 含HER2的异二聚体介导有力的丝裂原信号, 他比其他家族成员结合更多的磷酪氨酸结合蛋白亚群; 由于与生长因子缓慢解离率, 他的异源二聚体比其他异源二聚体有更高的配基亲和力和特异性^[12, 15]^。虽然HER2或HER3均可被配体激活, 目前认为, HER2/HER3异源二聚体是HER家族中相互作用最强的一对, 介导配体诱导的酪氨酸磷酸化以及下游信号传递^[15, 16]^。

## 2 HER2在NSCLC中的过表达

在包括NSCLC在内的不同实体瘤患者中, 均发现了HER2的过表达。虽然HER2是乳腺癌患者很好的预测和预后指标, 但在肺癌中的作用却还不明确。

### 2.1 HER2过表达的检测

免疫组化(immunohistochemistry, IHC)是检测HER2过表达最常使用的方法。报道中NSCLC患者HER2过表达的频率变化幅度很大。这些频率差异可能是由于方法学上的差异以及被研究的患者群体的不同。HER2过表达的总体频率, 在IHC换算2+/3+, 范围为4.3%^[17, 18]^-34.9%^[19]^。

在NSCLC中, 通过IHC和荧光原位杂交技术(fluorescence *in situ* hybridization, FISH)分析, HER2过表达和*HER2*扩增之间有重叠, 在IHC3+的肿瘤中表现尤为明显^[17, 19-22]^。虽然也有报道IHC阴性或低染色时FISH染色阳性^[20-23]^, 但许多原因可以加以解释, 比如固定和组织处理的质量以及转录或转录后调控机制的影响; 在IHC2+的肿瘤中, 免疫结果阳性FISH阴性的可能原因是自分泌环及突变形成而不是基因扩增^[21, 24]^。

除了IHC测定法, 其他作者还试图使用其他方法评估HER2过表达, 如实时聚合酶链式反应(real time-polymerase chain reaction, RT-PCR)。Brabender等^[25]^使用肺肿瘤和正常组织之比1.8为临界值, 报告了HER2高mRNA水平与预后不良有关, HER2/EGFR共表达对生存的影响是叠加的(*P*=0.176)。另一研究^[26]^报道了Ⅰ期NSCLC患者中HER2及EGFR共表达与更短的无病存活率(*P*=0.006)和总生存率(*P*=0.027)相关。EGFR和HER2在NSCLC的同步过表达作用被评价为预测吉非替尼作用的生物标记, 这表明基于这些受体的双重靶向治疗可为特定的患者带来益处^[27, 28]^。此外, Vallbohmer等^[29]^使用RT-PCR技术, 发现90例可切除的原发NSCLC患者*HER2*基因表达水平与存活时间相关, HER2低表达水平的女性与高表达女性相比具有明显长的生存期, 但这仅在女性中, 男性中却未发现差异。这种明显的性别差异的分子机制目前还不得而知。

### 2.2 HER2过表达的预后作用

有3项不同的*meta*分析^[30-32]^报道了HER2过表达的不良预后。最近的一篇^[32]^分析了40项此类研究, 共纳入6, 135例患者, 显示HER2过表达与NSCLC不良预后明显相关(危险比1.48), 甚至在疾病的早期阶段。当作者将分析限制在鳞状细胞癌时, 他们并未发现任何预后效果。但是, 这个结果需要慎重解释, 因为这些研究中存在着内在差异, 如选择的差异、数据获取的差异以及年龄、性别等因素对结果的影响。。

HER2阳性NSCLC患者具有较短总生存期的一个可能原因是这些肿瘤内在的化疗抗性^[33]^。Tsai等^[34]^通过Northern印记分析, 首次报道了*HER2*基因表达的NSCLC细胞系对包括卡铂在内的6种常用化疗药物存在内在抗性。此外, 他们将*HER2*基因转染到低表达p185的NSCLC细胞系中, 极大增强了转染后细胞系的化疗抵抗^[35]^, 同时未经治疗的NSCLC细胞系, 当表达高水平p185时表现出相似的化疗抗性^[36]^。Junker等^[37]^通过IHC和FISH对Ⅲa N2及Ⅲb期NSCLC患者手术前后标本的HER2状态进行评价, 表达HER2的肿瘤表现出较低的治疗引起的肿瘤消退趋势, 这表明HER2阳性肿瘤对化疗和放疗有相对的抵抗性。相反, 有报道^[38, 39]^显示, 无论是FISH或者IHC检测, HER2状况都与局部晚期NSCLC对放化疗的反应无关。另一研究^[40]^评估了*EGFR*和*HER2*基因拷贝数对一线化疗的预测价值, 在FISH阳性和FISH阴性的晚期NSCLC患者中未发现对化疗反应方面的任何差异(*P*=0.5);然而, FISH检测HER2阳性患者中, EGFR-TKIs作为二线或三线治疗, 有较高的缓解率(response rate, RR)、进展所需时间(time to progression, TTP)和总生存时间(overall survival, OS), 而在HER2 FISH检测阴性的患者中, 化疗产生更高RR和更长TTP。

### 2.3 HER2过表达的预测作用

由于HER2是EGFR首选的结合体, 临床前数据^[41-46]^表明, HER2过表达的细胞对吉非替尼敏感, 一些学者评估HER2失调对EGFR-TKIs敏感性的影响。在*EGFR*突变发现前, Cappuzzo等^[19]^通过IHC在63例未选择的吉非替尼治疗的NSCLC患者中, 评估了HER2过表达的影响。在43例可评估的患者中, EGFR和HER2阳性的频率分别是55.8%和34.8%(18.6% IHC3+)。但不论HER2阳性抑或HER2/EGFR过表达, 在TTP或OS方面都无影响。据Noberasco等^[18]^报道, 一个类似的患者群体, 在23例可评价EGFR和HER2的过表达患者中, 使用吉非替尼治疗后, 11例患者部分缓解(partial response, PR)/疾病稳定(stable disease, SD), 12例患者病情进展(progressive disease, PD)。有趣的是, 只有HER2 IHC3+的患者获得PR, 3个具有低染色(IHC1+)的患者获得PD。这将极有助于预测未选择的患者对吉非替尼的敏感性。然而HER2 IHC3+患者的相对少见, EGFR-TKI敏感突变的发现, 限制了这方便的研究。

由于NSCLC患者的抗HER2药物曲妥珠单抗的使用, HER2过度表达的预测作用得到了越来越广泛的研究。鉴于曲妥珠单抗在HER2阳性乳腺癌的治疗中的明确作用, 以及在表达HER2的NSCLC细胞系中, 单独使用或者联合化疗药物(与吉西他滨具有最大协同作用)产生良好的临床前活性证据^[47]^, 对其在具有HER2活性的晚期NSCLC中进行了研究, 这些研究中, HER2活性变化标准:IHC(2+/3+), 通过FISH进行*HER2*扩增, 或通过酶联免疫吸附试验(enzyme-linked immunosorbent assay, ELISA)检测HER2血清水平(> 15 ng/mL)。总的来说, 这些研究没有证明曲妥珠单抗联合紫杉烷类药物, 或作为单一药物使用的显著优势^[48-52]^。尽管结果有些令人失望, 限制了对HER2高表达或扩增患者的研究, 但也出现了曲妥珠单抗联合铂类的建议^[48, 49]^, 这一方向还需要进一步的研究。

## 3 *HER2*在NSCLC中的基因扩增 

众所周知, 基因扩增是原癌基因发挥作用的机制, 在多种人类恶性肿瘤包括肺部肿瘤中存在。然而, 与乳腺癌相比, NSCLC中*HER2*扩增似乎很不常见, 更频繁是17号染色体多体形成^[21]^。

### 3.1 *HER2*扩增的检测

已有多种方法用来评估NSCLC *HER2*基因组的获得。有一个普遍的共识是, FISH基因/染色体的比率大于2, 代表真正的基因扩增。然而, 其他参数也常被使用^[21]^, 因此, 不同的研究中检测频率相差很大。

### 3.2 *HER2*扩增的预测作用

在一些研究中^[27, 28, 53, 54]^, *HER2*扩增与*EGFR*突变有关, 确切机制不明。相反, Endo等^[55]^的研究并未发现*HER2*扩增与*EGFR*突变相关。Pugh等^[56]^发现, HER2 FISH阳性与*EGFR*突变或者扩增无相关性。

Cappuzzo等^[27]^评估了NSCLC中, *HER2*扩增预测吉非替尼治疗敏感性。23例HER2 FISH阳性的患者(22.8%), 有更高的总缓解率(overall response rate, ORR)、疾病控制率(disease control rate, DCR)及更长TTP。其中, 19例进行了*EGFR*突变的检测, 8例含有*EGFR*突变, 但只有1例对吉非替尼有反应。11例HER2 FISH阳性EGFR阴性的患者, 3例SD, 8例PD。7例HER2 FISH阳性EGFR阳性的患者对治疗有反应。双阳性患者治疗效果明显好于双阴性或者单阴性的患者。在7例FISH HER2阴性EGFR阳性的患者中, 只有1例表现出客观反应, 这组患者与EGFR阴性组的患者有着相似的结果, 表明*HER2*基因的高表达增加了吉非替尼治疗的敏感性。相反, 缺乏EGFR、HER2单独存在并不能驱动吉非替尼的敏感性。Daniele等^[28]^证实了这一结果, 他们的报道表明只有在*HER2*及*EGFR*扩增同时发生时吉非替尼才有敏感性。

### 3.3 *HER2*扩增作为EGFR-TKIs耐药的新机制

*HER2*扩增被认为是结直肠癌患者对抗EGFR的单抗隆抗体西妥昔单抗产生获得性耐药的一个机制^[57, 58]^, HER2家族信号的活性与ALK抑制剂的获得性耐药密切相关^[59]^, 这表明受体酪氨酸激酶信号的交替激活(旁路途径)是逃避的靶向药物的共同机制。近日, Takezawa等^[60]^在*EGFR*突变HER2异常的NSCLC细胞系中进行研究, 基于临床前和临床数据中, 阿法替尼(抑制EGFR、HER2、HER4)和西妥昔单抗两药联合在EGFR-TKIs获得性耐药模型中的效果, 他们首次报道了*HER2*扩增作为NSCLC患者中EGFR-TKIs耐药的新机制, 在不存在T790M突变的情况下12%患者产生耐药。T790M突变是公认的NSCLC患者中EGFR-TKIs耐药的机制, 耐药率达49%-62%^[61, 62]^。这些至少部分解释了, 没有T790M介导的获得性耐药, 为何阿法替尼和西妥昔单抗双阻滞所诱导的反应发生在了一部分而不是所有患者身上^[60]^。在这些*HER2*扩增肿瘤中, 没有可用于HER2 FISH分析的治疗前标本, 以排除TKIs使用前*HER2*扩增的存在。然而, 他们检测了99例未处理腺癌标本, *HER2*扩增仅占1例(1%)。

Yu等^[62]^证实了这些结果。155例*EGFR*突变的NSCLC患者中, 获得EGFR-TKIs抗药后进行重新活检, 4%的患者中有不同抗药机制的相互重叠, 1例患者同时存在着T790M和*HER2*扩增, 这表明不同的肿瘤克隆中可能存在着不同的耐药机制, 进一步说明了获得性耐药的异质性。不幸的是, 他们没能在治疗前标本和所有再活检标本(155例仅24例检测了*HER2*扩增)进行HER2 FISH检测, 使得难以得出一个明确结论:有关这些获得性遗传变异及重叠机制的真正的比率。

由于NSCLC中*HER2*基因异常的频率较低, 评估抗HER2药物曲妥珠单抗的活性的试验还处于Ⅱ阶段。然而, Gatzemeier等^[49]^将曲妥珠单抗与顺铂及吉西他滨联合, 与HER2低表达/FISH阴性的患者相比, 少数HER2高表达和/或扩增的患者表现出较高的RR和较长的无进展生存期(progression-free survival, PFS)。这表明在HER2高表达和/或扩增的患者中, 三药联合带来了益处。*HER2*扩增作为EGFR-TKIs获得性耐药机制之一的发现, 重燃了曲妥珠单抗在NSCLC中的研究, 以及在*EGFR*突变、使用EGFR-TKIs后进展、活检发现HER2阳性的患者中进行的Ⅱ阶段研究(NCT02226757)。拉帕替尼是EGFR和HER2的共同抑制剂, 在非选择性NSCLC群体的Ⅱ期临床试验中表现出极低的单药疗效。然而, *HER2*扩增的患者可能从拉法替尼中获益, 2例具有*HER2*突变的患者中, 1例肿瘤体积缩小51%^[62]^。

## 4 *HER2*在NSCLC中的基因突变

### 4.1 *HER2*突变

2004年, Stephens等^[63]^首次报道了在NSCLC中存在着*HER2*酪氨酸激酶结构域的突变。在120例NSCLC样本中进行测序发现, 4.2%样本在激酶结构域发生四个框内突变和一个碱基错义替换, 这与EGFR框内缺失类似, 这些遗传的改变与已知的促癌因子(*EGFR*/*KRAS2*/*NRAS*/*BRAF*)相互排斥。随后Mazières等^[64]^报道了有些样本中含有其他促癌因子。

体外研究^[65-68]^表明*HER2*外显子20突变导致了其不依赖于配体的基本活性, 信号转导活性强于HER2WT。更重要的是他们对肿瘤的维持很重要, 并且对EGFR-TKIs不敏感, 但是仍然对HER2靶向药物敏感。

紧随Stephens等^[63]^的报道之后, 其他组织陆续发现在人类多种恶性肿瘤中存在*HER2*突变, 包括乳腺癌^[69, 70]^、直肠癌^[71]^、膀胱上皮癌^[72]^。在这些研究中发现, 在非肺肿瘤*HER2*突变的类型和定位与NSCLC有很大区别, 有更高的错义突变, 而不是缺失/插入, 涉及并不是外显子20。这表明*HER2*突变的功能机制在其他肿瘤与肺癌中可能有所不同。

*HER2*突变的出现定义了NSCLC的一个分子亚群, 这个亚群具有特殊的临床病理特征:亚裔^[73]^、女性^[64, 74, 75]^、不吸烟^[73, 74, 76, 77]^、腺癌^[73, 76]^、甚至具有细支气管肺泡特征^[78]^、高分化^[77]^、TTF-1染色阳性^[74]^、特殊的临床表现^[64]^, 与其他分子亚群相比类似的生存率^[77]^。虽然有着特殊的亚群特征, *HER2*突变也可以在男性以及过度吸烟者中发现, 表明HER2检测应该通过组织学而不是临床特征^[64, 77]^。最近, Barlesi等^[78]^在一大型分子标记研究中发现, 10, 000例晚期白人NSCLC患者中*HER2*突变率占0.9%。

在一些患者中, *HER2*突变的发生与*HER2*及*EGFR*拷贝数的增加有关。实际上, 在Li等^[75]^的研究中, 8例*HER2*突变的样本中, 7例*HER2*拷贝数增加, 5例*EGFR*拷贝数增加。然而, 大样本中并没有发现*HER2*突变与基因扩增相关^[77]^, 二者的关系有待进一步研究。

最近, Yamamoto等^[79]^在多发肺癌的日本家族中发现了新型*HER2*突变(G660D)。通过全外显子测序, 他们首次证明了HER2跨膜结构域的种系突变, 推测HER2跨膜结构域突变可能扮演了驱动子突变。315例NSCLC样品中*HER2*基因17外显子的测序证明, 1例非粘液腺原位癌患者中存在一个额外的跨膜突变(V659E)。人类V659E突变与老鼠V664E突变类似。V664E被认为促进了致癌转化。实际上, 他们本可以发表最初数据以支持其作为促癌启动子作用。

p95HER2, 已知的HER2羧基端片段, 是HER2受体一个缩短了的亚型, 缺乏细胞外结构域重要部分, 其在大部分乳腺癌中存在, 导致对曲妥珠单抗的抵抗^[80]^。Cappuzzo等^[81]^发现p95 HER2在不到10% NSCLC患者中存在, 同时大部分没有*HER2*扩增和突变, 并没有预后价值。

### 4.2 HER2抑制剂在*HER2*突变患者中的临床应用

临床前研究表明*HER2*突变的肿瘤对EGFR-TKIs相对不敏感^[65-67]^, 尽管可能由于HER2表达的水平不同导致不敏感的水平不同^[82]^。这些数据在临床上得到确认, 因为含有*HER2*突变的NSCLC患者对吉非替尼没反应^[83, 84]^或稍许反应^[74]^。

2006年, Cappuzzo等^[10]^首次报道了1例60岁、不吸烟、女性转移性肺腺癌患者, 对传统化疗药物及EGFR-TKIs吉非替尼均不敏感, 每周曲妥珠单抗联合紫杉醇治疗, 产生了部分反应, 对组织样本的分析揭示了外显子20突变的存在。其他作者^[74]^也报道了*HER2*突变患者使用曲妥珠单抗联合长春瑞滨治疗获益。

最近, Falchook等^[85]^在Ⅰ阶段的试验中, 对*HER2*外显子20插入突变的患者, 采用双重HER2抑制剂曲妥珠单抗和拉帕替尼联合抗血管生成药贝伐珠单抗, 产生了令人惊叹的效果。

临床前数据^[66, 82]^表明, 不可逆的泛HER抑制剂奈拉替尼抑制了绝大部分*HER2*突变体, 在类似的程度上强于*EGFR* L858R突变细胞系。最近, Gandhi等^[86]^报道了在Ⅰ期研究中, 奈拉替尼与mTOR抑制剂坦西莫斯两药联合具有抗肿瘤活性, 需要进一步评估。同时表明在HER2促发的NSCLC中, 存在*HER2*外显子20突变依赖的Akt/mTOR的信号通道。一个在HER2扩增乳腺癌及*HER2*突变NSCLC中评估这个联合作用的Ⅱ期研究正在进行(NCT01827267)。

Perera等^[67]^在*HER2*突变NSCLC中, 通过与mTOR抑制剂雷帕霉素联用, 首次报道了泛HER抑制剂阿法替尼临床前的效果。De Grève等^[87]^报道了阿法替尼在含有HER2外显子20突变的患者体内起作用的证据。这些患者先前使用了多种化疗药物及抗EGFR和/或抗HER2药物。

另一个不可逆的泛HER抑制剂dacomitinib, 在含有*EGFR*、*HER2*突变及*HER2*扩增的NSCLC细胞系的体外研究中具有一定作用。Kelly等^[88]^报道1例*EGFR*和*HER2*野生型双表达的患者, 在曲妥珠单抗和长春瑞滨联合治疗进展后, 使用dacomitinib治疗PR。晚期NSCLC患者, *HER2*突变或扩增的亚群中使用dacomitinib治疗Ⅱ期临床试验的初步结果已见诸报道^[89]^。在*HER2*突变的NSCLC, 报道了3个月PFS及10个月的OS, ORR达13%(对*HER2*外显子20插入突变起反应, 2例*HER2*点突变的患者并没观察到反应)。相反, 在*HER2*扩增的患者中无反应。PFS介于1个月-5个月。

最近, Mazières等^[64]^报道了目前为止样本量最大的*HER2*突变同期NSCLC患者, 使用多种抗HER2药物后的治疗效果。在22例可评估的患者中, ORR达50%, DCR达82%, PFS达5.1个月。他们还发现曲妥珠单抗联合化疗药物的DCR达96%, 阿法替尼单药达到100%, 拉法替尼和马赛替尼单药治疗无反应。然而, 抗HER2药物治疗患者的群体限制性以及治疗的特异性并不能得出一个有关*HER2*突变在NSCLC中的预后和预测作用决定性的结论。在选择性临床分子治疗方面却有着预期价值。

NSCLC的生物标记物法国国家计划^[78]^和肺癌突变联盟等多种生物标记物平台的利用, 可能有助于识别和治疗患者这些罕见的异常。近日, 美国国家癌症研究所开展了一系列临床研究, 总体目标是用更精确的诊断, 让患者选择适合的有针对性的治疗。美国国家癌症研究所的MATCH研究将瞄准下一代测序技术介导的分子异常, 对“MATCH”患者进行适当的靶向药物治疗, 这是一个多分子参与的Ⅱ期临床研究^[90]^。NSCLC研究迫切需要创新性和主动性, MATCH试验可能有助于分子靶向药物更广泛地利用, 即使是临床上分子亚型罕见的群体。

## 5 总结与展望

目前为止, HER2在NSCLC中的作用还不是很清晰。然而, *HER2*突变的发现, 临床上抗HER2药物作用的证实, 重燃了NSCLC中HER2作用的研究。NSCLC中抗HER2治疗的重视, 为不可逆的泛HER抑制剂的临床使用指明了方向; *HER2*扩增作为EGFR-TKIs获得性耐药新机制还需要进一步的研究, 这可为临床中靶向HER2提供依据; 此外, *HER2*扩增/过表达可否作为NSCLC预测和/或预后指标, 从而影响到临床个体化治疗的决策, 这还需要进一步探讨。期待HER2在NSCLC患者个体化治疗中有光明的未来。
